
JOURNAL INFORMATION
==============================
NLM Title Abbreviation: Ultrasound Obstet Gynecol
Journal ID: Ultrasound Obstet Gynecol
NLM Journal ID: 9108340
Journal ID: UOG

Ultrasound in Obstetrics & Gynecology

ISSN: 0960-7692
EISSN: 1469-0705

ARTICLE INFORMATION
==============================
PMCID: PMC9350292
PMID: 35809244
DOI: 10.1002/uog.26029
Article ID: UOG26029
Article ID: UOG-2022-0325.R2
Article version: 1
Subjects: Letter to the Editor, Letters to the Editor

Fetal death and placental lesions after two COVID‐19 episodes in the same pregnancy in an unvaccinated woman

Two COVID‐19 episodes in one same pregnancy

Dubucs et al.

Dubucs C. https://orcid.org/0000-0003-2371-4113 1 2 charlotte@iuct-oncopole.fr dubucs.charlotte@iuct-oncopole.fr

Groussolles M. 2
Brazet E. 3
Courtade‐Saïdi M. 1
Van Acker N. 4
Ousselin J. 1
Pasquier C. 5
Aziza J. 1
1 Department of Cytology and Pathology IUCT‐Oncopole Toulouse France
2 SPHERE ‐ Equipe constitutive du CERPOP, UMR 1295, unité mixte de recherche Inserm – Université Toulouse III Paul Sabatier
3 Gyneco‐obstetrics Department Paule de Viguier Maternity Hospital, CHU Toulouse France
4 Department of Cytology and Pathology CHU, Imag'IN Platform, IUCT‐Oncopole Toulouse France
5 Virology department Institute Federative of biology, CHU Toulouse France
* Correspondence to: C. Dubucs, dubucs.charlotte@iuct-oncopole.fr Department of Cytology and Pathology, IUCT‐Oncopole, Toulouse, France
Electronic publication date: 2022 Jul 9
Electronic Location ID: 10.1002/uog.26029
Revised 2022 Jun 30; Received 2022 Apr 14; Accepted 2022 Jul 1
Copyright: This article is protected by copyright. All rights reserved.
License: This is an open access article under the terms of the http://creativecommons.org/licenses/by/4.0/ License, which permits use, distribution and reproduction in any medium, provided the original work is properly cited.
License URL: https://creativecommons.org/licenses/by/4.0/

Figure count: 0
Table count: 0
Page count: 1

source-schema-version-number: 2.0
edited-state: accepted-manuscript
details-of-publishers-convertor: Converter:WILEY_ML3GV2_TO_JATSPMC version:6.2.0 mode:remove_FC converted:12.10.2022

This article has been accepted for publication and undergone full peer review but has not been through the copyediting, typesetting, pagination and proofreading process, which may lead to differences between this version and the Version of Record. Please cite this article as doi: 10.1002/uog.26029.

==============================

Dubucs Charlotte (Orcid ID: 0000-0003-2371-4113)

### Fetal death and placental lesions after two COVID-19 episodes in the same pregnancy in an unvaccinated woman

C. Dubucs[1,2], M. Groussolles[2], E. Brazet[3], M. Courtade-Saïdi[1], N. Van Acker[4], J. Ousselin[1], C. Pasquier[5], J. Aziza[1]

1. Department of Cytology and Pathology, IUCT-Oncopole, Toulouse, France
2. SPHERE - Equipe constitutive du CERPOP, UMR 1295, unité mixte de recherche Inserm – Université Toulouse III Paul Sabatier
3. Gyneco-obstetrics Department, Paule de Viguier Maternity Hospital, CHU Toulouse, France
4. Department of Cytology and Pathology, CHU, Imag'IN Platform, IUCT-Oncopole, Toulouse, France
5. Virology department, Institute Federative of biology, CHU Toulouse, France

Correspondence to: C. Dubucs, dubucs.charlotte@iuct-oncopole.fr

Department of Cytology and Pathology, IUCT-Oncopole, Toulouse, France

Accepted Article

This article has been accepted for publication and undergone full peer review but has not been through the copyediting, typesetting, pagination and proofreading process which may lead to differences between this version and the Version of Record. Please cite this article as doi: 10.1002/uog.26029

This article is protected by copyright. All rights reserved.

Schwartz et al. showed that maternal SARS-CoV-2 infection can generate specific and severe histopathological placental lesions [1]. According to Kotlyar et al., around 20% of SARS-CoV-2 positive unvaccinated mothers show placental lesions with positive immunohistochemistry for SARS-CoV-2 in villous trophoblast [2]. SARS-CoV-2 placental lesions can be involved in intrauterine fetal death by functional placental insufficiency when placental damage is massive associating trophoblastic necrosis, perivillous fibrin deposition, chronic intervillositis as well as extensive hemorrhagic changes [3, 4]. In Dubucs and colleagues' paper, intrauterine fetal death (IUFD) occurred between 1 to 3 weeks after positive SARS-CoV-2 maternal RT-PCR testing and is observed in 2nd and 3rd trimesters of pregnancies [5]. Severe SARS-CoV-2 placenta lesions occur despite mild maternal COVID-19 symptoms. In few cases, we found focal lesions that over time become less inflammatory, richer in fibrin deposits with only focal or even negative immunostaining for SARS-CoV-2 making the histological diagnosis of SARS-CoV-2 infection difficult [5]. We illustrate this statement with a case in which the mother developed COVID-19 two times during a same pregnancy.

The patient was not vaccinated for SARS-CoV-2, 33 years old with no medical history. The third pregnancy of the patient was marked by a first SARS-CoV-2 infection at 16+5WoG with mild maternal symptoms, mainly asthenia and dyspnea. The pregnancy had a normal evolution. The third trimester control was normal at 32+5WoG, with a fetal weight estimated at 2100g. At 33+4WoG she had a second SARS-CoV-2 infection with mild maternal symptoms. The clinical maternal tolerance was controlled every 48h. At day 9 post-infection, the patient became symptomatic with fever and influenza-like syndrome. Forty-eight hours later, she was clinically stable with antipyretics and hydration. Ultrasound and clinical control showed fetal biometrics at 75$^{th}$ percentile, normal fetal cardiac rhythm and amniotic fluid quantity, with absence of doppler anomalies.

Unfortunately, the pregnancy outcome led to IUFD at 36+6WoG. The standard IUFD exams were negative: array CGH, CMV and Parvovirus B19 research in amniotic fluid, as well as maternal autoimmune tests excluding other causes of IUFD. She gave birth to a stillborn baby boy with a birth weight of 2880g. Macroscopic examination of the placenta revealed abnormal cross-sectional slices showing highly heterogeneous and hemorrhagic sections of the parenchyma. Histological exam of her placenta showed extensive lesional areas characterized by trophoblast necrosis associated with confluent perivillous fibrinous deposits and inflammatory histiocytic lymphocytic infiltrate (CD3+/CD68+) mixed with cellular debris in the intervillous chamber, suggestive of both ancient and recent placental lesions (Figures 1 and 2). Interestingly, immunohistochemical study with anti-SARS-CoV-2 nucleocapsid antibody,

Accepted Article shows heterogeneous staining with (i) diffuse and intense labeling of cytotrophoblast and villous syncytiotrophoblast in lesional areas; (ii) the absence of labeling in non-lesioned areas; (iii) and the absence of labeling in some areas of trophoblast necrosis, raising the question of possible old sequelae.

These organized lesions linked to SARS-CoV-2 but with only focally positive or negative SARS-CoV-2 immunochemistry highlights the fact that lesions become negative over time. Indeed, the SARS-CoV-2 variant Delta was found by RT-PCR in lesioned areas whether positive or not with anti-SARS-CoV-2 antibody. Moreover, the fact that two successive maternal COVID-19 infections occurred during one same pregnancy could suggest that immunization might not be systematically acquired during pregnancy with Delta variant and highlights significant immune evasion capabilities of this variant.

*Ethics/consent statement: This paper support the principles of the Declaration of Helsinki, and ethical consent are not required for this purpose. All patient informations are anonymous.

Accepted Article

Bibliography

1. Schwartz DA, Morotti D. Placental Pathology of COVID-19 with and without Fetal and Neonatal Infection: Trophoblast Necrosis and Chronic Histiocytic Intervillositis as Risk Factors for Transplacental Transmission of SARS-CoV-2. *Viruses* 2020; 12.

2. Kotlyar AM, Grechukhina O, Chen A, Popkhadze S, Grimshaw A, Tal O, Taylor HS, Tal R. Vertical transmission of coronavirus disease 2019: a systematic review and meta-analysis. *Am J Obstet Gynecol* 2021; 224: 35-53 e33.

3. Schwartz DA, Avvad-Portari E, Babal P, Baldewijns M, Blomberg M, Bouachba A, Camacho J, Collardeau-Frachon S, Colson A, Dehaene I, Ferreres JC, Fitzgerald B, Garrido-Pontnou M, Gerges H, Hargitai B, Helguera-Repetto AC, Holmstrom S, Irles CL, Leijonhfvud A, Libbrecht S, Marton T, McEntagart N, Molina JT, Morotti R, Nadal A, Navarro A, Nelander M, Oviedo A, Oyamada Otani AR, Papadogiannakis N, Petersen AC, Roberts DJ, Saad AG, Sand A, Schoenmakers S, Sehn JK, Simpson PR, Thomas K, Valdespino-Vazquez MY, van der Meeren LE, Van Dorpe J, Verdijk RM, Watkins JC, Zaigham M. Placental Tissue Destruction and Insufficiency from COVID-19 Causes Stillbirth and Neonatal Death from Hypoxic-Ischemic Injury: A Study of 68 Cases with SARS-CoV-2 Placentitis from 12 Countries. *Arch Pathol Lab Med* 2022. DOI: 10.5858/arpa.2022-0029-SA.

4. Schwartz DA. Stillbirth after COVID-19 in Unvaccinated Mothers Can Result from SARS-CoV-2 Placentitis, Placental Insufficiency, and Hypoxic Ischemic Fetal Demise, Not Direct Fetal Infection: Potential Role of Maternal Vaccination in Pregnancy. *Viruses* 2022; 14.

5. Dubucs C, Groussolles M, Ousselin J, Sartor A, Van Acker N, Vayssiere C, Pasquier C, Reyre J, Batlle L, Favarel Clinical Research Associate S, Duchanois Midwife D, Jauffret Clinical Research Associate V, Courtade-Saidi M, Aziza J. Severe placental lesions due to maternal SARS-CoV-2 infection associated to intrauterine fetal death. *Hum Pathol* 2022; 121: 46-55.

Accepted Article

Figure 1: Image suggestive of recent lesions. A) Immunohistochemistry (IHC) using anti SARS-CoV-2 nucleocapsid antibody (clone bsb-134 diagomics france): Intense brown staining at the periphery of the chorionic villi representative of trophoblast infection. B) Hematoxyline and eosin (HE) staining: Intervillous hemorrhagic lesions associated with extensive trophoblast necrosis and chronic intervillitis (lympho- histiocytic infiltrate).

Figure 2: images suggestive of old sequellar lesions: A) IHC with anti-SARS-CoV-2 nucleocapsid antibody showing negativity of the villous trophoblast in contrast to recent lesions showed in figure 1. B) Massive perivillous fibrin deposition with villous infarctions and without both hemorrhage and significant inflammatory infiltrate suggestive of a possible sequellar lesion (HE).

Accepted Article

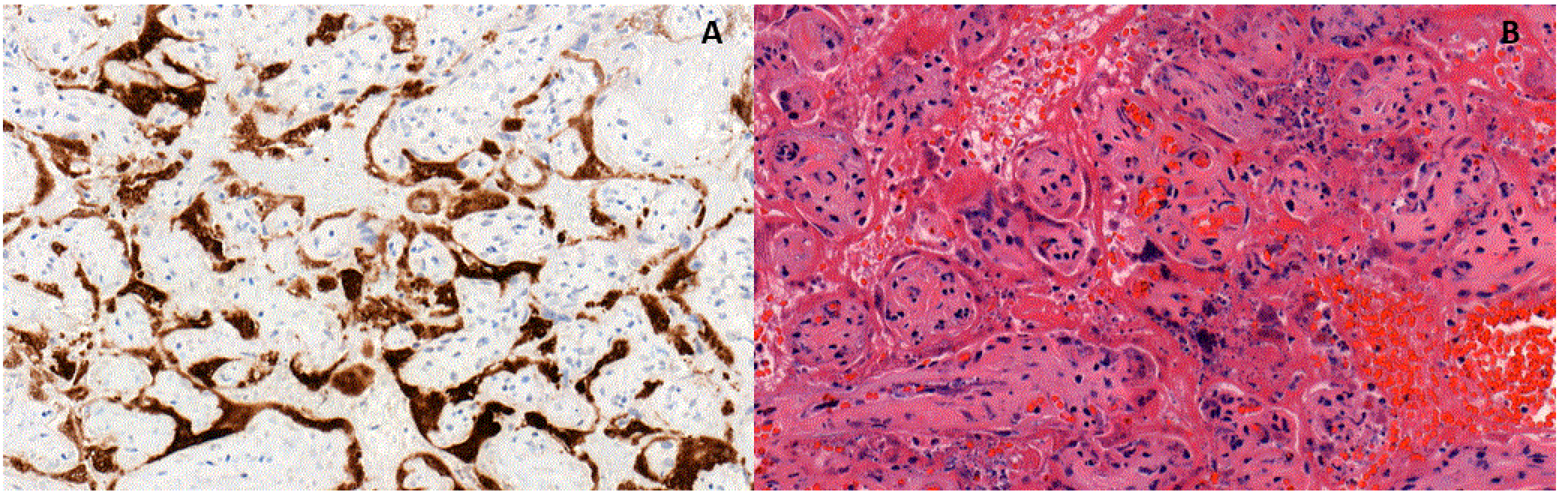

UOG_26029_Figure 1.GIF

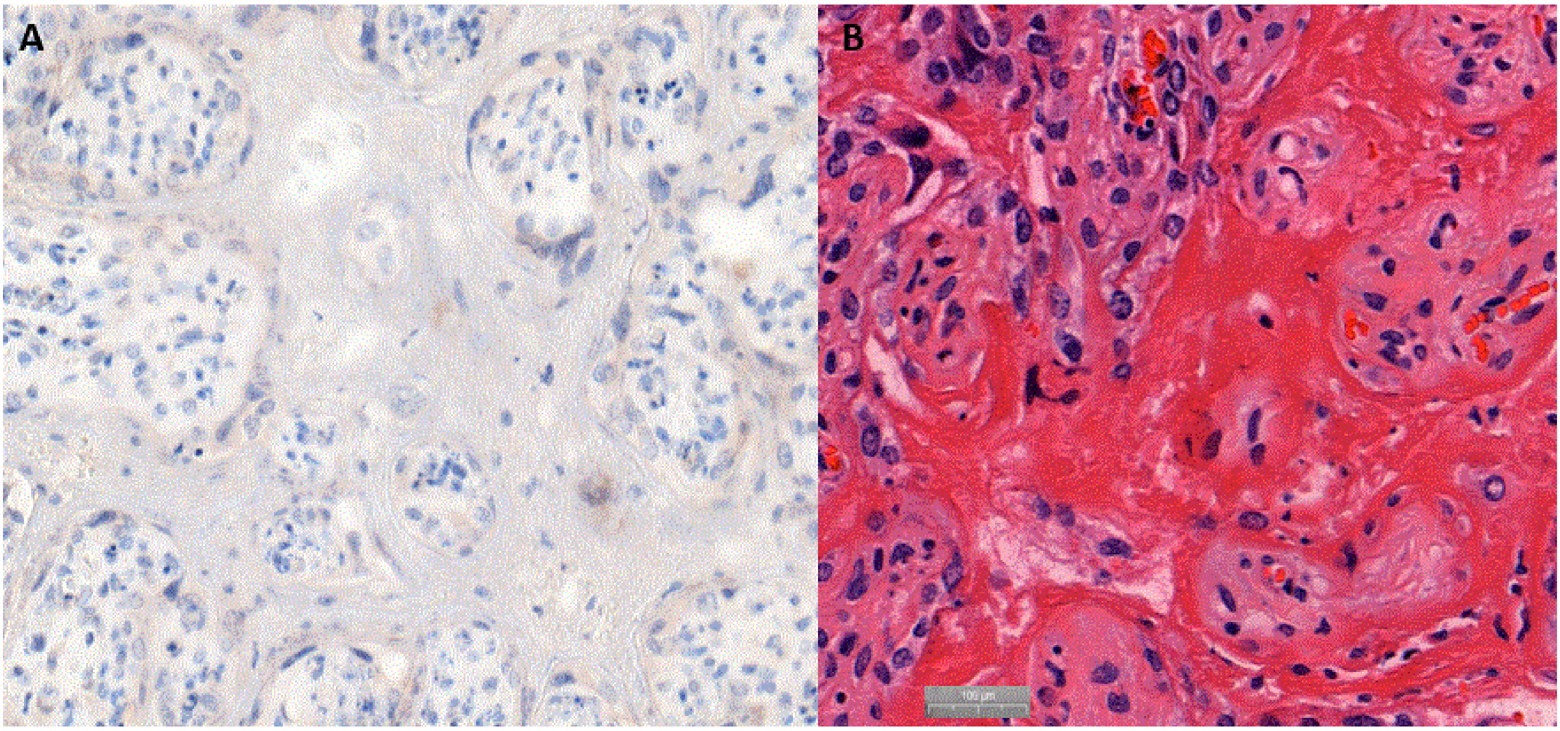

UOG_26029_Figure 2.GIF
